# Giant Cervicothoracic Ganglioneuroma Misdiagnosed as Neurofibroma on Core Needle Biopsy: Transcervical Resection and Postoperative Neurological Sequelae: A Case Report

**DOI:** 10.1002/ccr3.73816

**Published:** 2026-10-29

**Authors:** Omar Daas, Somaya Al Kiswani, Mohammad Z. Al‐Qaisi, Ahmad Daas, Ashjan Al Shakkah, Sohaib Salahat, Abdullah Nofal

**Affiliations:** ^1^ Department of Medicine The University of Jordan Amman Jordan; ^2^ Radiology Department King Hussein Cancer Center Amman Jordan; ^3^ Radiology Department Hamad Medical Corporation Doha Qatar; ^4^ Department of Neurosurgery Ibn al‐Haytham Hospital Amman Jordan; ^5^ Oncology Imaging Fellow, Radiology Department King Hussein Cancer Center Amman Jordan; ^6^ Department of Medicine, Faculty of Medicine and Health Sciences An‐Najah National University Nablus Palestine; ^7^ Head of Diagnostic Radiology Department King Hussein Cancer Center Amman Jordan

**Keywords:** cervicothoracic, ganglioneuroma, Horner syndrome, neurofibroma, transcervical, vocal cord paralysis

## Abstract

Ganglioneuroma (GN) may be misdiagnosed as neurofibroma when core needle biopsy samples only contain Schwannian stroma without mature ganglion cells. Even giant cervicothoracic GNs may be resectable through a transcervical approach alone. Recurrent laryngeal nerve palsy and Horner syndrome are foreseeable, anatomically predictable risks requiring dedicated preoperative counseling.

## 1 Introduction

Ganglioneuroma (GN) is a benign, fully differentiated tumor of sympathetic neural crest origin, histologically characterized by mature or maturing ganglion cells intermixed with Schwann cells and fibrous stroma [1, 2]. It represents the most differentiated, benign end of the neuroblastic tumor spectrum, which also encompasses neuroblastoma and ganglioneuroblastoma; unlike these malignant counterparts, GN lacks neuroblasts, mitotic activity, and necrosis [1]. Although histologically benign, GN can attain substantial size before clinical detection and, depending on its anatomical location, may exert significant mass effect on adjacent vital structures, which makes its recognition clinically important even in the absence of malignant potential.

GN most commonly arises in the posterior mediastinum, retroperitoneum, or adrenal gland, with thoracic and abdominal nonadrenal sites predominating. It typically affects older children and young adults, with a reported median age of approximately 7 years, although cases in adults are well documented [1]. Cervical GN is uncommon, and cervicothoracic extension across the thoracic inlet is extremely rare [3, 4]. No specific environmental or hereditary risk factors have been consistently implicated in GN, and, unlike neurofibroma, it is not characteristically associated with neurofibromatosis. Most tumors are asymptomatic and discovered incidentally; when symptomatic, mass effect may produce pain, dysphagia, dyspnea, or neurological deficits, including Horner syndrome or vocal cord paralysis [1, 2, 5]. Because of overlapping imaging and cytological features, the principal differential diagnoses for a cervicothoracic neurogenic mass include neurofibroma, schwannoma, and, particularly in younger patients, neuroblastic tumors such as ganglioneuroblastoma.

Preoperative diagnosis is challenging: cross‐sectional imaging findings are nonspecific, and core needle biopsy may sample only Schwannian stroma while missing the sparse, diagnostic ganglion cells, particularly within large, heterogeneous tumors [6]. Ding et al. and Lee et al. each reported GNs that were initially misdiagnosed as neurofibroma on needle biopsy, underscoring the potential for discordance between limited tissue sampling and final surgical pathology [3, 7]. Definitive diagnosis, therefore, generally requires examination of the fully resected specimen; immunohistochemical positivity for S100 and SOX10 supports a neurogenic origin but does not reliably distinguish GN from other peripheral nerve sheath tumors when only limited tissue is available [6, 7]. Surgical excision is the mainstay of treatment and is generally curative, but it is technically demanding in the cervicothoracic region owing to proximity to the carotid and subclavian arteries and brachial plexus [8]; combined cervical and thoracoscopic approaches have been described for thoracic‐outlet lesions [9, 10]. Prognosis following resection is excellent. A recent nationwide study reported favorable long‐term outcomes despite incomplete resection in patients with GN and ganglioneuroblastoma intermixed; however, because these represent distinct pathological entities, caution is warranted when extrapolating these findings to pure GN [11].

We present a case of a 26‐year‐old woman with a giant cervicothoracic GN misdiagnosed as a neurofibroma on core needle biopsy, resected en bloc through a transcervical approach alone. GN is a well‐recognized benign neurogenic tumor, particularly among posterior mediastinal tumors, and its occurrence in this location is not, by itself, unusual. However, cervical involvement with extension across the thoracic inlet into the superior mediastinum is uncommon. The clinical significance of the present case lies in the unusual cervicothoracic extent, the discrepancy between the initial core biopsy and the definitive resection diagnosis, and the technical challenges of surgical resection in this anatomically constrained region.

## 2 Case Presentation

A 26‐year‐old woman with no significant past medical or surgical history and no personal or family history of neurofibromatosis or other hereditary cancer predisposition presented in May 2025 after incidental detection of a right upper thoracic mass on imaging performed for an unrelated indication. She was a non‐smoker with an Eastern Cooperative Oncology Group performance status of 0. Her chief complaint was mild dysphagia. On presentation, vital signs were stable, and physical examination was otherwise unremarkable.

### 2.1 Differential Diagnosis, Investigations, and Treatment

Contrast‐enhanced CT demonstrated a large, well‐defined, low‐attenuation right upper mediastinal mass extending through the thoracic inlet (Figure 1A–E). PET/CT was subsequently obtained to characterize the metabolic activity of the lesion and screen for FDG‐avid nodal or distant disease, given that a malignant neurogenic or lymphoproliferative process could not be excluded on CT alone. Dedicated MRI, which could have offered superior soft‐tissue and nerve‐sheath delineation, particularly at the tracheoesophageal groove and cervical sympathetic chain, was not obtained because it was not logistically feasible within the required preoperative timeframe at our institution. PET/CT showed mild FDG uptake within the mass (Figure 1F–H) and mildly FDG‐avid thoracic lymph nodes, findings consistent with a benign or reactive process. CT‐guided Tru‐Cut core needle biopsy revealed a moderately cellular spindle‐cell lesion with wavy nuclei, without necrosis or mitotic activity. Immunohistochemistry was positive for S100 and SOX10 and negative for beta‐catenin, smooth muscle actin, desmin, and CD34. Based on these findings, the differential diagnosis was narrowed to a benign peripheral nerve sheath tumor, and the lesion was interpreted as consistent with neurofibroma; schwannoma was considered less likely given the biopsy pattern, and malignant peripheral nerve sheath tumor was considered unlikely given the absence of necrosis, mitotic activity, and significant FDG avidity. GN was not favored preoperatively, as the biopsy had not captured the ganglion cell component required for this diagnosis.

**FIGURE 1:**
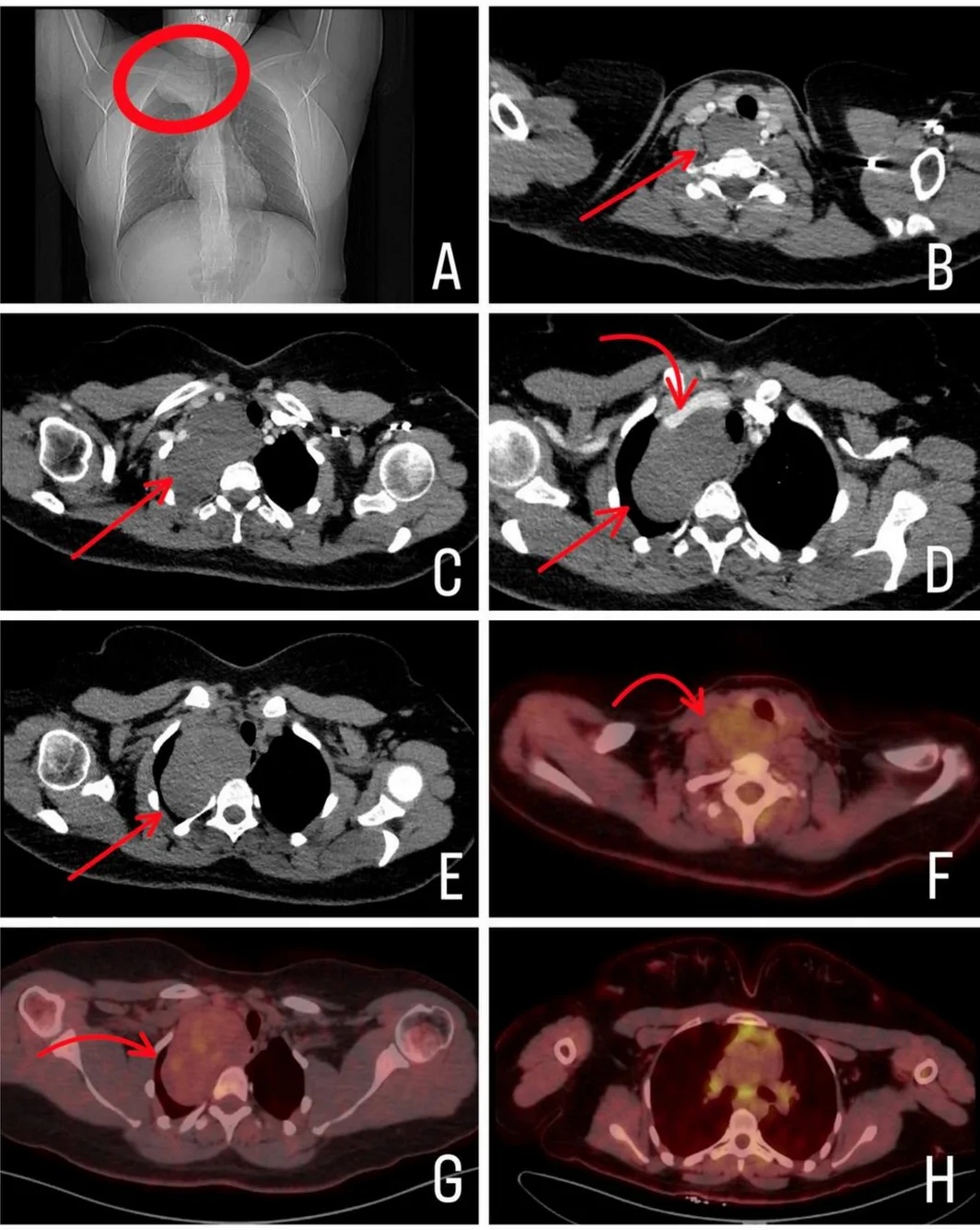
Preoperative CT and PET/CT imaging of the neck and chest. (A) Scout CT image demonstrating a large right cervicothoracic soft tissue mass (red circle) causing leftward tracheal deviation. (B–E) Serial axial contrast‐enhanced CT images showing a well‐circumscribed hypodense mass (red arrows) extending from the lower neck through the thoracic inlet into the superior mediastinum, with mass effect on the carotid sheath and adjacent great vessels. (F–H) Axial fused PET/CT images demonstrating mild heterogeneous fluorodeoxyglucose (FDG) uptake within the lesion (red curved arrows), consistent with a benign neurogenic tumor.

Pulmonary function testing showed an FEV1 of 90% predicted and a DLCO of 125% predicted. Echocardiography demonstrated preserved biventricular function. The case was reviewed at the institutional Sarcoma Multidisciplinary Clinic; given the tumor's proximity to the trachea, esophagus, innominate artery, and right carotid artery, the operative goal was defined as safe maximal resection rather than mandatory complete (R0) excision. Informed consent was obtained after counseling regarding vascular, airway, neurologic, and sympathetic chain risks.

In July 2025, the patient underwent right neck exploration under general anesthesia in the supine position. An oblique incision was made along the right sternocleidomastoid muscle. After division of the platysma and development of the plane between the strap muscles, the tumor was identified posteromedial to the right carotid artery. Sharp and blunt dissection allowed mobilization of the mass from the carotid artery, innominate artery, esophagus, and trachea, and multiple feeding vessels were ligated. The mass was delivered en bloc through the cervical incision alone, without sternotomy, thoracotomy, clavicular division, or thoracoscopic assistance. A Jackson‐Pratt drain was placed, and the wound was closed in layers. The remainder of the immediate postoperative period was uneventful, and the patient was discharged on postoperative Day 2.

The resected specimen measured 12.0 × 8.0 × 5.0 cm, with a tan, homogeneous cut surface and focal myxoid change. Histological examination demonstrated mature ganglion cells embedded within Schwannian stroma, without evidence of necrosis or mitotic activity, thereby establishing the diagnosis of GN. The resection margin was microscopically positive at the deep aspect abutting the tracheoesophageal groove, where the tumor was intimately adherent to the recurrent laryngeal nerve. The preoperative interpretation of neurofibroma was attributed to sampling of S100/SOX10‐positive Schwannian spindle‐cell stroma on the core biopsy without capture of the mature ganglion cells required for a definitive diagnosis of GN (Table 1).

**TABLE 1:** Comparison of histopathological and immunohistochemical findings between the initial core needle biopsy and definitive resection specimen.

Feature	Core needle biopsy (Preoperative)	Resection specimen (Definitive)
Tissue sampled	Limited core(s) from a small area of a large, heterogeneous mass	Entire tumor, 12.0 × 8.0 × 5.0 cm, tan and homogeneous with focal myxoid change
Cellular pattern	Moderately cellular spindle‐cell lesion with wavy nuclei	Mature ganglion cells embedded within Schwannian spindle‐cell stroma
Ganglion cells	Not identified	Present and diagnostic
Necrosis/mitoses	Absent	Absent
Immunohistochemistry	S100 (+), SOX10 (+), beta‐catenin (−), SMA (−), desmin (−), CD34 (−)	Not repeated; findings consistent with neurogenic origin already established on biopsy
Working/final diagnosis	Benign peripheral nerve sheath tumor, favored as neurofibroma	Ganglioneuroma

On postoperative Day 1, the patient developed hoarseness; fiberoptic laryngoscopy confirmed right vocal cord paralysis with a patent airway. She subsequently reported dysphagia, a globus sensation, chronic dry cough, and aspiration with thin liquids. Right eye ptosis with reactive pupils also developed, consistent with postoperative Horner syndrome. In late July 2025, a 5 cm soft swelling appeared lateral to the surgical wound; CT confirmed a postoperative seroma, which resolved spontaneously without intervention. At 3 months postoperatively, swallowing had improved by approximately 70%–80%. Follow‐up CT in October 2025 and January 2026 demonstrated no residual or recurrent disease (Figure 2).

**FIGURE 2:**
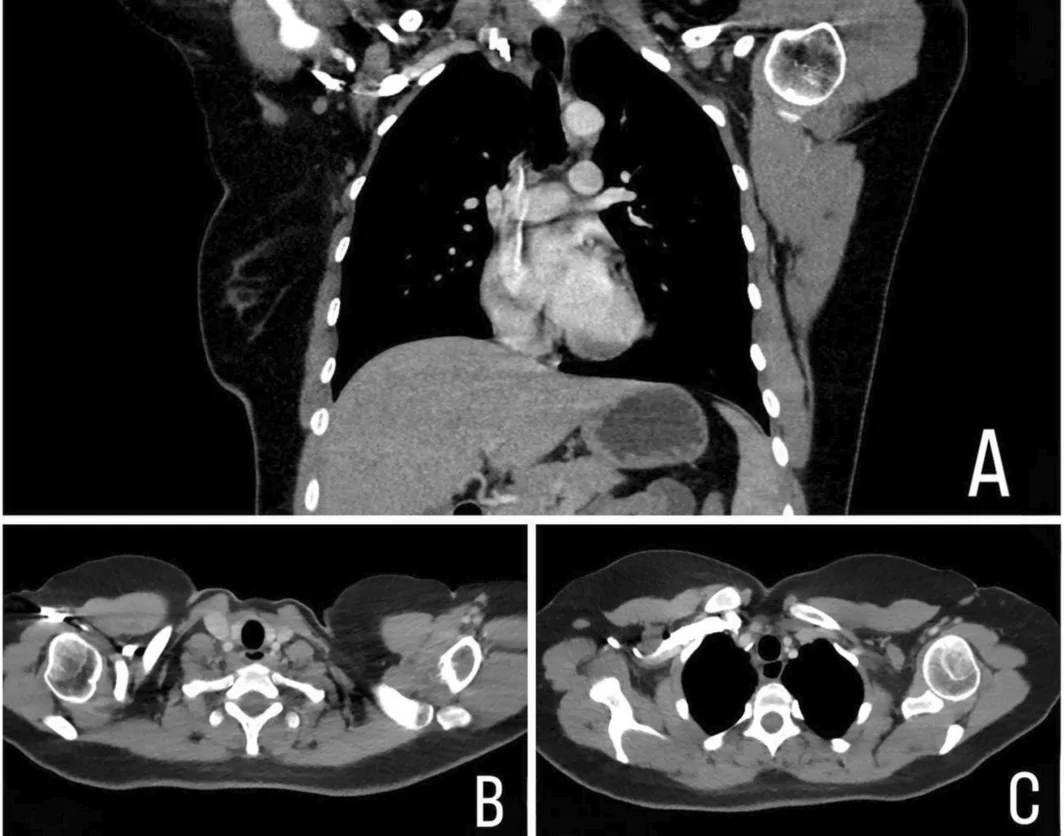
Follow‐up CT of the neck and chest at 6 months post‐resection. (A) Coronal view demonstrating postoperative changes along the right thoracic inlet with complete removal of the mass and no gross recurrence. (B, C) Serial axial view slices confirming anatomical restoration and absence of residual tumor in the tracheoesophageal groove and upper mediastinum.

At the final clinic visit in February 2026, repeat laryngoscopy showed persistent right vocal cord paralysis with full contralateral compensation, near‐normal voice, and resolution of aspiration. Ptosis had improved but had not fully resolved. Surveillance imaging every four to 6 months was planned. At the last follow‐up, the patient remained clinically stable with no evidence of local recurrence and satisfactory functional compensation of her postoperative neurological deficits (Table 2).

**TABLE 2:** Clinical course and timeline of a patient with giant cervicothoracic ganglioneuroma.

Date/Time	Clinical course	Key investigations/Findings	Management/Outcome
May 2025	Incidental right upper thoracic mass; mild dysphagia	CT: large right upper mediastinal mass extending through the thoracic inlet	Further evaluation initiated
Preoperative 2025	Diagnostic work‐up	PET/CT: mild FDG uptake. Core biopsy: S100/SOX10‐positive spindle‐cell lesion, interpreted as neurofibroma	Multidisciplinary review and surgical planning
July 2025	Definitive treatment	Tumor closely related to carotid artery, innominate artery, trachea, and esophagus	En bloc transcervical resection; discharged on postoperative day 2
July 2025	Definitive diagnosis	Resection specimen: mature ganglion cells within Schwannian stroma; no necrosis or mitotic activity	**Ganglioneuroma diagnosed.** Deep margin microscopically positive
Postoperative period	Hoarseness, dysphagia, aspiration, and right ptosis	Laryngoscopy: right vocal cord paralysis; findings consistent with Horner syndrome. CT: postoperative seroma	Seroma resolved spontaneously; neurological deficits monitored
3 months postoperatively	Functional improvement	—	Swallowing improved by approximately 70%–80%
October 2025–January 2026	Surveillance	CT showed no residual or recurrent disease	Continued surveillance
February 2026	Final reported follow‐up	Persistent right vocal cord paralysis with contralateral compensation; improved ptosis	Near‐normal voice, resolved aspiration, no evidence of recurrence; surveillance every 4–6 months planned

*Note:* Bold value shows the point at which the definite diagnosis was established.

## 3 Conclusion

This case demonstrates that GN may be misclassified as neurofibroma on core needle biopsy when mature ganglion cells are not captured, highlighting an important diagnostic limitation of limited tissue sampling. It also shows that a transcervical‐only approach can achieve safe resection of selected large cervicothoracic GNs while avoiding sternotomy or thoracotomy. Finally, recurrent laryngeal nerve dysfunction and Horner syndrome should be recognized as foreseeable consequences of surgery in this region and incorporated into preoperative counseling. Reporting technically challenging cases such as this may help refine surgical decision‐making and improve patient counseling for these rare tumors.

## 4 Discussion

GN is a benign, fully differentiated neurogenic tumor that, despite its innocuous histology, can pose substantial diagnostic and surgical challenges when it arises in an anatomically constrained region such as the cervicothoracic junction. The present case illustrates three findings of practical relevance to clinicians managing neurogenic masses in this location: the propensity for core needle biopsy to misrepresent the true diagnosis, the feasibility of complete transcervical resection even for a giant tumor with mediastinal extension, and the anatomically predictable, rather than idiosyncratic, nature of the postoperative neurological deficits that followed.

Epidemiologically, GN is a tumor of childhood and adolescence, with a reported median age at diagnosis of approximately 7 years and a strong predilection for the posterior mediastinum, retroperitoneum, and adrenal gland [1]. Cervical involvement accounts for a small minority of cases, and extension across the thoracic inlet into the superior mediastinum, as seen in our patient, is distinctly uncommon, with only isolated reports in the literature [3, 4]. Our patient's age of 26 years is therefore atypical relative to the usual pediatric and adolescent distribution, a divergence also noted in other adult case reports of cervical and mediastinal GN [3, 8]. This reinforces that, although classically a disease of the young, GN should remain within the differential diagnosis of a cervicothoracic neurogenic mass in adults, and that age alone should not be used to discount the diagnosis.

Pathophysiologically, GN arises from the same sympathetic neural crest lineage as neuroblastoma and ganglioneuroblastoma but occupies the fully matured, non‐proliferative end of this spectrum, containing mature ganglion cells, Schwann cells, and fibrous stroma without neuroblasts, mitoses, or necrosis [1, 2]. This maturity accounts for its indolent behavior and explains why, unlike its malignant counterparts, GN rarely causes systemic symptoms and instead produces disease through local mass effect. In our patient, this mass effect manifested as dysphagia, the only presenting symptom, consistent with the typical incidental or mildly symptomatic presentation described in the literature, in which pain, dyspnea, or compressive neurological deficits such as Horner syndrome or vocal cord paralysis may also occur, depending on the structures involved [1, 2, 5]. The absence of more dramatic symptoms despite a 12 cm tumor is itself notable and underscores the slow, expansile growth pattern characteristic of this entity.

The diagnostic pathway in this case exemplifies a recognized pitfall in the pre‐surgical evaluation of GN: the discordance between core needle biopsy and final surgical pathology. Cross‐sectional imaging, while useful for delineating tumor extent and relationship to adjacent structures, is inherently nonspecific for tissue diagnosis; the well‐defined, low‐attenuation appearance and mild FDG avidity seen on our patient's CT and PET/CT were compatible with a benign neurogenic tumor but could not distinguish GN from neurofibroma or schwannoma [12]. Nonetheless, imaging remained essential to surgical planning: contrast‐enhanced CT delineated the tumor's relationship to the carotid and innominate arteries, trachea, and esophagus, informing the decision to pursue a cervical‐only surgical approach, while the absence of marked FDG avidity on PET/CT reduced concern for a malignant neurogenic or neuroblastic tumor and supported proceeding with resection without further oncologic work‐up. Dedicated MRI was not obtained in this patient because it was not logistically feasible within the required preoperative timeframe; MRI's superior soft‐tissue contrast may have better delineated the tumor's relationship to the recurrent laryngeal nerve and cervical sympathetic chain and could be considered where available in similar future cases, although it would not have resolved the underlying histological ambiguity addressed by biopsy. The core biopsy sampled S100‐ and SOX10‐positive Schwannian spindle cells without any ganglion cell component, leading to a preoperative diagnosis of neurofibroma. This scenario parallels the experience of Ding et al. and Lee et al., who each described GNs initially misread as neurofibroma on needle biopsy because the ganglion cells, often sparse and unevenly distributed within a bulky tumor, were simply not represented in the sampled core [3, 7]. Bapir et al. similarly emphasized that the immunohistochemical profile of GN overlaps substantially with other peripheral nerve sheath tumors on limited tissue, since S100 and SOX10 positivity confirms a neurogenic origin but does not, by itself, establish the presence of mature ganglion cells [6]. Taken together, these observations support a general principle: in a large, heterogeneous cervicothoracic neurogenic mass, a core biopsy diagnosis of neurofibroma should be interpreted with caution, and clinicians should be prepared for the final diagnosis to change once the entire specimen is available for histological examination.

The differential diagnosis in this case reasonably included neurofibroma, schwannoma, and, given the patient's presentation, malignant peripheral nerve sheath tumor and neuroblastic tumors such as ganglioneuroblastoma. Schwannoma was considered less likely because of the specific spindle‐cell pattern on biopsy, while the absence of necrosis, mitotic activity, and marked FDG avidity argued against a malignant peripheral nerve sheath tumor or an immature neuroblastic tumor. This reasoning was sound, given the available preoperative data; however, it also illustrates the central limitation of biopsy‐based differential diagnosis in this tumor group, namely that the distinction between GN and neurofibroma ultimately rests on identifying mature ganglion cells, a feature that a small‐volume biopsy is poorly equipped to capture. The case therefore reinforces that imaging and biopsy narrow the differential diagnosis but do not substitute for examination of the resected specimen when treatment planning and prognosis depend on the specific neurogenic tumor subtype.

From a treatment perspective, surgical excision remains the accepted standard for GN, and outcomes following resection are generally excellent regardless of the completeness of margins [11]. The principal challenge in the cervicothoracic region is not whether to operate, but how to gain safe access to a tumor that spans two anatomically distinct compartments connected by a narrow, vascular, and neurologically dense corridor. Several groups have described combined cervical and thoracoscopic, or anterior transcervicothoracic, approaches to manage this challenge, reasoning that dual‐compartment exposure reduces the risk of injury during mobilization of the intrathoracic component [9, 10]. Our case adds to a smaller body of evidence indicating that, in appropriately selected patients, a cervicothoracic GN of substantial size and inlet‐spanning extent can instead be resected entirely through a cervical incision, without recourse to sternotomy, thoracotomy, or thoracoscopy. This distinction is clinically meaningful because it avoids the additional morbidity, longer recovery, and cosmetic consequences associated with an intrathoracic incision, provided the tumor's plane relative to the great vessels and airway permits safe cervical‐only mobilization, as was confirmed intraoperatively in this patient.

The surgical considerations in this case also merit scrutiny with respect to margin status and neurological outcome. In our patient, a microscopically positive margin was intentionally accepted to avoid recurrent laryngeal nerve injury. This approach was based on the benign biology of GN and supported, albeit indirectly, by evidence from a large United Kingdom nationwide cohort demonstrating excellent outcomes after incomplete resection of GN and ganglioneuroblastoma intermixed [11]. Because that cohort included two distinct pathological entities, these findings should be interpreted cautiously when applied to pure GN. Nevertheless, preserving critical neurovascular structures may justify accepting microscopic residual disease when complete excision would entail substantial morbidity. Serial imaging through January 2026 in our patient showed no residual or recurrent disease, providing early reassurance that this trade‐off was justified, although continued surveillance remains warranted given the relatively short follow‐up interval to date.

The postoperative course, marked by vocal cord paralysis and Horner syndrome, should be understood as an anatomically coherent consequence of dissection in this region rather than an unusual complication. Vocal cord paralysis reflects the recurrent laryngeal nerve's course through the tracheoesophageal groove, precisely where the tumor was most adherent, and comparable vocal cord dysfunction has been reported after cervical GN resection by Bassami et al. and by Cannady et al. in their series of pediatric cervical GN [5, 13]. Horner syndrome similarly reflects disruption of the cervical sympathetic chain, a recognized risk whenever dissection extends along the paravertebral or carotid sheath region, as also described by Gary et al. in a retropharyngeal GN resection [14]. What distinguishes this case is less the occurrence of these deficits than their trajectory: vocal cord paralysis persisted but was functionally compensated, with near‐normal voice and resolved aspiration, and ptosis partially improved, indicating that even permanent structural nerve injury in this region can be associated with a favorable functional outcome through contralateral and central compensation.

This report has several limitations. Intraoperative photographs were not obtained during surgery, and the archival core biopsy material could not be retrieved, so photomicrographs of the biopsy and resection specimens are unavailable; these findings are therefore described in text and summarized in Table 1 rather than illustrated. Dedicated MRI was also not performed for logistical reasons. Finally, as a single case with short follow‐up, the findings may not be generalizable, and continued surveillance is warranted.

## Author Contributions

**Omar Daas:** writing – original draft, writing – review and editing, data curation. **Somaya Al Kiswani:** writing – original draft, writing – review and editing, data curation. **Mohammad Z. Al‐Qaisi:** writing – original draft, writing – review and editing, data curation. **Ahmad Daas:** writing – original draft, writing – review and editing, data curation. **Ashjan Al Shakkah:** writing – original draft, writing – review and editing, data curation. **Sohaib Salahat:** writing – original draft, writing – review and editing. **Abdullah Nofal:** writing – original draft, writing – review and editing, data curation.

## Funding

The authors have nothing to report.

## Ethics Statement

Our institutional review board does not require ethical approval for single case reports.

## Consent

Written informed consent from the patient was obtained for publication according to journal guidelines.

## Conflicts of Interest

The authors declare no conflicts of interest.

## Data Availability

Data sharing not applicable to this article as no datasets were generated or analyzed during the current study.
